# Audit of Quality and Effectiveness of Clinical Note Entries on a Women’s Low/Medium Secure Forensic Psychiatry Inpatient Unit

**DOI:** 10.1192/bjo.2025.10574

**Published:** 2025-06-20

**Authors:** John Dillon

**Affiliations:** Norfolk and Suffolk NHS Foundation Trust, Norwich, United Kingdom

## Abstract

**Aims:** This audit aims to assess adherence to the ‘CRAMS’ criteria in clinical records for patients on a blended low/medium secure women’s forensic ward. In response to feedback from a CQC Mental Health Act Review that some daily entries in patient notes included insufficient detail to properly inform decisions on care (particularly with regard to qualitative information regarding leave), the service adopted the CRAMS criteria for these entries, which specify that information on 11 points relating to care plan, risk, activity, mental state, and Section 17 leave should be included.

**Methods:** Data were collected from daily electronic patient record entries for ten randomly selected patients on two different weeks – in total 140 entries were examined in this initial sample. Each of these entries was assessed to determine if it contained each of the 11 aspects of the CRAMS criteria.

Following the initial data collection, findings were disseminated and discussed with the team, an information sheet was produced, and CRAMS was discussed at daily team huddles with the aim of improving adherence. A further sample of 140 records was examined a year following the initial cycle, using the same methodology.

**Results:** The initial cycle of the audit found that from the 140 clinical note entries that were examined, a total of 814/1540 (52.9%) of CRAMS criteria were met. This included:

212/420 (50.5%) criteria pertaining to care planning.

99/140 (70.7%) criteria pertaining to risk.

153/280 (54.6%) criteria pertaining to activity undertaken.

209/420 (49.8%) criteria pertaining to mental state.

141/280 (50.3%) criteria pertaining to S17 leave.

The second cycle of the audit found that a total of 1120/1540 (71.6%) of CRAMS criteria were met. This included:

305/420 (72.6%) criteria pertaining to care planning.

105/140 (75.0%) criteria pertaining to risk.

198/280 (70.7%) criteria pertaining to activity undertaken.

296/420 (70.5%) criteria pertaining to mental state.

198/280 (70.7%) criteria pertaining to S17 leave.

**Conclusion:** The audit showed a significant improvement in adherence to the CRAMS criteria following the action plan enacted following the initial cycle. However, there were still numerous entries in which the full CRAMS criteria were not met. The ward team continues to discuss CRAMS as part of morning briefings to sustain and build upon this improvement, and the adoption of an electronic template to help further build on this once the Trust moves to a new electronic patient record system has been discussed.

